# Adalimumab effectively reduces the signs and symptoms of active ankylosing spondylitis in patients with total spinal ankylosis

**DOI:** 10.1136/ard.2007.082529

**Published:** 2007-12-04

**Authors:** D van der Heijde, A L Pangan, M H Schiff, J Braun, M Borofsky, J Torre, J C Davis, R L Wong, H Kupper, E Collantes, for the ATLAS Study Group

**Affiliations:** 1 Leiden University Medical Center, Leiden, The Netherlands; 2 Abbott Laboratories, Abbott Park, Illinois, USA; 3 Denver Arthritis Clinic Research Unit, Denver, Colorado, USA; 4 Ruhr University Bochum, Bochum, Germany; 5 Arthritis and Osteoporosis Center, Clinical Research Center of Reading, West Reading, Pennsylvania, USA; 6 Hospital Monte Naranco, Oviedo-Asturias, Spain; 7 University of California, San Francisco, California, USA; 8 Abbott Laboratories, Parsippany, New Jersey, USA; 9 Abbott GmbH & Co. KG, Ludwigshafen, Germany; 10 Hospital Universitario Reina Sofia, Cordova, Spain

## Abstract

**Objective:**

To evaluate the long-term safety and efficacy of adalimumab in patients with ankylosing spondylitis (AS) and total spinal ankylosis (TSA).

**Design:**

Patients (n = 315) with active AS were randomised in a 2:1 ratio to receive adalimumab 40 mg every other week or placebo for 24 weeks followed by open-label adalimumab for up to 5 years. Two-year efficacy and safety data for 11 patients with investigator-defined TSA were evaluated. The primary end point was the ASsessment in AS International Working Group criteria for 20% improvement (ASAS20) at Week 12. On or after Week 12, ASAS20 non-responders could switch to open-label adalimumab. Other efficacy measurements included ASAS40, ASAS 5/6, ASAS partial remission, and 50% improvement in the Bath AS Disease Activity Index (BASDAI 50).

**Results:**

6 of 11 TSA patients were randomised to adalimumab and 5 to placebo. At Week 12, 50% of the adalimumab-treated patients achieved an ASAS20 response and 33% achieved an ASAS40, ASAS 5/6 and BASDAI 50. No placebo-treated patients achieved any response criteria at Week 12. 4 placebo- and 2 adalimumab-treated patients switched to open-label adalimumab before Week 24. After 1 year of adalimumab treatment, 8 of 11 patients achieved an ASAS20 response. After 2 years, 6 of the remaining 8 patients with TSA reported an ASAS20 response. There were no serious adverse events or adverse event-related study discontinuations.

**Conclusion:**

In patients with TSA, adalimumab treatment resulted in rapid and clinically significant improvement in the signs and symptoms of active disease. Adalimumab effectiveness and safety were sustained for at least 2 years.

**Trial registration number:**

NCT00085644.

Ankylosing spondylitis (AS) typically strikes young adults, with the burden of disease attributable primarily to the resulting functional disability.^1^ The disease course varies widely. Some patients experience sacroiliitis alone, while others experience rapid progression to end-stage fusion of the spine, or total spinal ankylosis (TSA).^2^ Patients who develop TSA (ie, bamboo spine) experience significantly more functional impairment and are less likely to be employed compared with other patients with AS.^3^ In addition to substantial functional disability, patients with TSA may experience a more debilitating disease course. The fragility of the rigid spinal column increases the risk of spinal fractures and possible neurological sequelae, and spinal deformities may contribute to respiratory and other difficulties.^1^ In contrast to pre-existing concepts, patients with TSA may continue to have signs and symptoms of active AS, which are insufficiently responsive to non-steroidal anti-inflammatory drugs (NSAIDs).

Patients with TSA are typically excluded from participation in randomised controlled trials of therapeutic agents for AS. For example, the randomised controlled trials of the tumour necrosis factor (TNF) antagonists etanercept and infliximab have excluded AS patients with TSA.^4^ ^5^ The Adalimumab Trial Evaluating Long-term Efficacy and Safety for AS (ATLAS) was the first large randomised controlled trial of a TNF antagonist in patients with active AS that permitted patients diagnosed with TSA.^6^ Our objective was to evaluate the long-term safety and efficacy of adalimumab in patients with TSA who had participated in ATLAS.

## PATIENTS AND METHODS

### Patients

ATLAS has been described in the published report of the 24-week, double-blind results.^6^ Adults with AS based on the modified New York criteria^7^ who had active disease were recruited for the study. ATLAS was designed with an a priori limit on enrolment of patients with TSA of 10%. A diagnosis of TSA was based on the investigators’ assessments of lateral radiographs of the cervical and lumbar spine and lateral views of chest radiographs. All enrolled patients had an inadequate response or intolerance of one or more NSAIDs, as defined by the investigators. Also, patients who had failed therapy with one or more disease-modifying antirheumatic drugs were allowed to participate.

Each of the 43 study centres obtained independent ethics committee approval, and ATLAS was conducted in accordance with the Declaration of Helsinki. Compliance with local laws and customs was assured by investigators at the 43 centres in Europe (Belgium, France, Germany, Italy, The Netherlands, Spain, Sweden, and the United Kingdom) and the USA. Written informed consent was obtained from each patient before any study-related procedures were initiated.

### Study design

Patients were randomised to receive adalimumab 40 mg every other week (eow) or matching placebo in a 2:1 ratio. Study medications were provided in prefilled syringes containing either adalimumab 40 mg or placebo for subcutaneous injection (Abbott Laboratories, Abbott Park, IL). The primary efficacy end point was the percentage of patients at Week 12 who achieved a 20% response according to the ASsessment in AS International Working Group criteria for improvement (ASAS20).^8^ Patients who did not achieve an ASAS20 response at Weeks 12, 16 or 20 were eligible to receive early escape, open-label treatment with adalimumab 40 mg eow. After the Week 24 visit, all patients were eligible to receive open-label adalimumab treatment in the ongoing study for up to 5 years. Data for patients with TSA who received adalimumab (blinded or open-label) for up to 2 years are presented here.

### Efficacy assessments

Additional efficacy assessments included the following criteria: ASAS40 response (defined as improvement of at least 40% and absolute improvement of at least two units (on a 0–10-point scale) compared with baseline in at least three of the four ASAS20 criteria domains with no deterioration in the remaining domain); ASAS 5/6 response (defined as at least 20% improvement in five of six of the following domains: the four domains of the ASAS20 criteria plus spinal mobility as measured by the 3-point Bath AS Metrology Index (BASMI)^9^ and an acute-phase reactant as measured by C-reactive protein concentration), and ASAS partial-remission response (defined as a value of <2 on a 0–10-point scale in each of the four ASAS20 domains).^10^ Mean values for each of the four ASAS20 criteria domains were also evaluated: patient’s global assessment of disease activity, total back pain, function according to the Bath AS Functional Index (BASFI),^11^ and inflammation based on the mean of questions 5 and 6 of the Bath AS Disease Activity Index (BASDAI) pertaining to morning stiffness severity and duration.^12^ An improvement of at least 50% in the BASDAI score (BASDAI 50), which measures the severity of fatigue, spinal and peripheral joint pain, local tenderness, and morning stiffness (both qualitative and quantitative) using a 0–10 cm visual analogue scale (VAS), was also noted for each patient.

### Safety assessments

Safety assessments were completed and adverse events were collected throughout the double-blind and open-label extension study periods. Flares of extra-articular disease manifestations were not prospectively or systematically collected during the study.

### Statistical analyses

Because of the small number of patients with TSA, there were no statistical comparisons made between treatment groups. Both efficacy and safety results are summarised descriptively. Observed data at Years 1 and 2 are based on total duration of adalimumab exposure (ie, double-blind and open-label treatment).

## RESULTS

### Baseline characteristics and study disposition

Of the 315 patients enrolled in ATLAS, 11 had investigator-diagnosed TSA; 5 were randomised to receive placebo and 6 were randomised to receive adalimumab 40 mg eow. Baseline demographics and disease activity for these 11 patients are summarised in table 1. After the Week 12 visit, 4 placebo-treated patients and 2 adalimumab-treated patients entered early escape, open-label treatment with adalimumab 40 mg eow. All 11 patients with TSA received 1 year of adalimumab treatment; 8 of these patients received at least 2 years of adalimumab treatment.

**Table 1:** Baseline demographic and clinical characteristics of the patients with total spinal ankylosis, by treatment group

Variable	Placebo (n = 5)	Adalimumab 40 mg eow (n = 6)
Male, n (%)	5 (100)	4 (66.7)
White, n (%)	5 (100)	6 (100)
Age (years), mean (SD)	50.2 (7.8)	54.2 (5.9)
Disease duration (years), mean (SD)	16.6 (5.9)	25.6 (7.4)
HLA-B27 positive, n (%)	5 (100)	4 (66.7)
DMARD use prior to or at baseline, n (%)	5 (100)	4 (66.7)
Concomitant NSAID use, n (%)	4 (80.0)	5 (83.3)
Concomitant corticosteroid use, n (%)	0 (0)	0 (0)
Patient’s global assessment of disease activity (cm), mean (SD)	6.2 (1.9)	8.7 (1.0)
Total back pain (cm), mean (SD)	5.1 (3.4)	6.6 (3.4)
Inflammation* (cm), mean (SD)	7.8 (1.7)	8.0 (0.8)
BASFI (cm), mean (SD)	7.5 (1.3)	8.0 (1.1)
BA(SD)AI (cm), mean (SD)	6.1 (1.6)	7.6 (1.0)
CRP (mg/dL), mean (SD)	2.9 (2.9)	3.3 (2.6)
BASMI (0–10), mean (SD)	6.4 (1.3)	7.8 (0.8)

BA(SD)AI, Bath Ankylosing Spondylitis Disease Activity Index; BASFI, Bath Ankylosing Spondylitis Functional Index; BASMI, Bath Ankylosing Spondylitis Metrology Index; CRP, C-reactive protein; DMARD, disease-modifying anti-rheumatic drug; eow, every other week; NSAID, non-steroidal anti-inflammatory drug.

*Mean of questions 5 and 6 of the BA(SD)AI.

### Efficacy

The ASAS20, ASAS40, ASAS 5/6, and BASDAI 50 responses for each patient with TSA are shown in table 2. At Week 12, all placebo-treated patients had no response based on any of these response criteria, whereas 50% of the six adalimumab-treated patients attained an ASAS20 response and 33% achieved an ASAS40, ASAS 5/6, and BASDAI 50 response. At Week 24, all four of the patients who remained on blinded adalimumab therapy had an ASAS20 response, two had an ASAS40 response, and three had an ASAS 5/6 and BASDAI 50 response. The one patient who continued to receive placebo at Week 24 did not achieve any of the response criteria. None of the patients with TSA in either treatment group achieved ASAS partial remission at Weeks 12 or 24.

**Table 2:** Efficacy results for patients with investigator-defined total spinal ankylosis or Stage V radiographic criteria during the 24-week, double-blind period

Treatment	Entered early escape therapy	Week 12 response				Week 24 response			
		ASAS20	ASAS40	ASAS 5/6	BASDAI 50	ASAS20	ASAS40	ASAS 5/6	BASDAI 50
ADA*	No	√	√	√	√	√	√	—	√
ADA	No	√	—	—	—	√	—	√	—
ADA	No	√	√	√	—	√	√	√	√
ADA*	No	—	—	—	√	√	—	√	√
ADA*	Yes	—	—	—	—	0	0	0	0
ADA*	Yes	—	—	—	—	0	0	0	0
PBO*	No	—	—	—	—	—	—	—	—
PBO	Yes	—	—	—	—	+	0	0	0
PBO*	Yes	—	—	—	—	+	+	+	+
PBO*	Yes	—	—	—	—	0	0	0	0
PBO*	Yes	—	—	—	—	+	+	+	+

ADA, adalimumab; ASAS, ASsessment in Ankylosing Spondylitis International Working Group criteria for improvement; BASDAI, Bath Ankylosing Spondylitis Disease Activity Index; PBO, placebo; √, Responder on blinded therapy; —, non-responder on blinded therapy; +, responder on early escape, open-label adalimumab; 0, non-responder on early escape, open-label adalimumab.

*Patients fulfilling Stage V radiographic criteria for TSA.

Of the four placebo-treated patients who switched to early escape, open-label adalimumab therapy on or after Week 12, three had an ASAS20 response and two had ASAS40, ASAS 5/6, and BASDAI 50 responses by Week 24.

After 1 year of adalimumab exposure, 8 of the 11 patients with TSA had an ASAS20 response, and 1 patient had ASAS-defined partial remission (table 3). After 2 years of exposure, there was continued clinical benefit from adalimumab therapy. The mean values for each of the four ASAS20 domains after 2 years of adalimumab exposure were: 3.5 for patient’s global assessment; 3.1 for total back pain; 4.9 for function (BASFI); and 3.8 for inflammation (items 5 and 6 of the BASDAI).

**Table 3:** Long-term efficacy results for patients with investigator-defined total spinal ankylosis or Stage V radiographic criteria*

Response variable	Investigator-defined TSA		Stage V TSA	
	Total duration of adalimumab exposure		Total duration of adalimumab exposure	
	1 year (n = 11), (%)	2 years (n = 8), (%)	1 year (n = 8), (%)	2 years (n = 5), (%)
ASAS20	72.7	75.0	62.5	60.0
ASAS40	36.4	62.5	37.5	40.0
ASAS 5/6	54.5	37.5	37.5	20.0
ASAS partial remission	9.1	0	12.5	0
BASDAI 50	45.5	62.5	50.0	60.0

ASAS, ASsessment in Ankylosing Spondylitis International Working Group criteria for improvement; BASDAI, Bath Ankylosing Spondylitis Disease Activity Index; TSA, total spinal ankylosis.

*All data are based on observed efficacy analyses.

After receiving adalimumab for 1 year, only one patient with TSA achieved ASAS partial remission; no patients were in partial remission after 2 years of exposure. This patient was the only one who had a value of <2 units (0–10 cm VAS) for function (BASFI). However, the following numbers of patients had values <2 units for the other ASAS domains after 1 year and 2 years of adalimumab treatment, respectively: patient’s global assessment, 3 and 2; total back pain, 4 and 4; and inflammation, 4 and 3.

There was minimal improvement in spinal mobility as measured by the BASMI. The observed mean changes in the BASMI for the adalimumab and placebo groups were 0.2 and 0.0 at Week 12 and −0.5 and 1.0 at Week 24, respectively. After 1 year of adalimumab treatment, the mean change in the BASMI was −0.6, and after 2 years, −0.8.

Braun and colleagues^2^ published a radiographic staging system for patients with AS. Stage V indicates widespread spinal ankylosis (ie, bamboo spine), defined as ⩾80% fusion of ⩾20 vertebrae. Using these staging criteria retrospectively, 8 of the 11 patients had Stage V ankylosis (4 in the placebo group and 4 in the adalimumab group). The remaining 3 patients had Stage IV radiographic changes, defined as radiographic evidence of spinal involvement in >2 segments (13–19 vertebrae, 50%–<80% of the spine). A post hoc analysis of these 8 Stage V patients revealed a similar response pattern compared with the 11 patients with investigator-defined TSA (tables 2 and 3).

### Safety

Complete safety data for all patients who participated in the randomised, controlled portion of ATLAS have been reported.^6^ All 11 patients reported at least one adverse event during the controlled or open-label periods, but none were serious and none led to discontinuation of study drug. There were no cases of opportunistic infections, tuberculosis, malignancies, congestive heart failure, demyelinating disorders, or lupus-like syndromes, and there were no deaths.

## DISCUSSION

Adalimumab therapy was associated with rapid and clinically significant improvement in the signs and symptoms of active AS in patients with TSA. In this small subgroup of patients with AS, it is remarkable that at Week 12, the time point for measurement of the primary efficacy end point, there was no placebo response based on either ASAS or BASDAI response criteria. The efficacy of adalimumab in patients with TSA was maintained through 2 years of treatment. No significant safety issues were observed in these patients.

The percentage of patients with TSA who experienced responses to adalimumab was similar to that reported for the overall study population of patients with active AS,^6 13^ except for ASAS-defined partial remission. ASAS-defined partial remission was achieved by only one patient with TSA (Stage V) after 1 year of adalimumab exposure. Although some patients had scores of <2 for one or more of the ASAS domains, only one patient was able to achieve this level for function (BASFI). In these patients who had a significant irreversible component of their disease status, certain response criteria may not appropriately reflect improvement in the reversible signs and symptoms of AS.

These results are novel and clinically relevant because ATLAS is the first and only randomised, controlled trial of a TNF antagonist to include patients with TSA.^4 5^ These patients have been excluded from participation in most clinical drug trials because it was likely assumed that they would experience minimal treatment benefit. However, this study demonstrated 1) that patients with TSA have evidence of active inflammatory disease; and 2) that the symptoms of active disease in these patients, including pain, functional impairment, and morning stiffness, can be substantially improved with adalimumab treatment. The overall pattern of response was similar when the results were analysed for patients who met specific radiographic staging criteria for TSA. Of particular clinical importance was the finding that patients with TSA who received long-term treatment with adalimumab (up to 2 years) have a sustained clinical response over time.

In conclusion, in patients with TSA, adalimumab therapy can result in rapid and sustained improvement in the signs and symptoms of active AS. Longer-term observations of these patients from the ATLAS trial will provide further insight on the potential benefit of anti-TNF therapy in this subgroup of patients with AS.
